# Moyamoya disease in Southeast Asians: genetic and autopsy data, new cases, systematic review, and meta-analysis of all patients from the literature

**DOI:** 10.1007/s00415-024-12228-0

**Published:** 2024-03-13

**Authors:** Daniel Strunk, Peter Bauer, Kathy Keyvani, Rolf R. Diehl, Roland Veltkamp, Peter Berlit, Sven G. Meuth, Lars Timmermann, Jan Claudius Schwitalla, Markus Kraemer

**Affiliations:** 1 Department of Neurology, Alfried Krupp Hospital, Alfried-Krupp-Straße 21, 45131 Essen, Germany; 2 Department of Neurology, University Hospital Gießen and Marburg, Marburg, Germany; 3 CENTOGENE GmbH, Rostock, Germany; 4 Institute of Neuropathology, University of Duisburg-Essen, Essen, Germany; 5 Department of Brain Sciences, Imperial College London, London, UK; 6 German Neurological Society, Berlin, Germany; 7 Department of Neurology, Medical Faculty, Heinrich-Heine University Düsseldorf, Düsseldorf, Germany; 8 Moyamoya Friends Association, Essen, Germany

**Keywords:** Moyamoya disease, *RNF213* p.R4810K variant, Histopathology, Southeast Asian, East Asian, European Caucasian

## Abstract

**Background:**

Moyamoya disease (MMD) is a rare disorder causing ischemic and hemorrhagic juvenile stroke. It is associated with the founder susceptibility variant p.R4810K in the *RNF213* gene in East Asia. Our aim was to enhance understanding of MMD in so far poorly characterized Southeast Asians and exploring differences with Caucasian Europeans.

**Methods:**

By retrospective analysis of medical records and systematic database search on PubMed for all published cases, we identified Southeast Asian patients with MMD. We extracted and pooled proportions using fixed-effects models. Our own cohort was tested for the East Asian *RNF213* founder variant p.R4810K. One of our Southeast Asian patients underwent post-mortem histopathological examination.

**Results:**

The study cohort comprised 32 Southeast Asians. Mean age at onset in the entire cohort was 32.5 ± 20.3 years (*n* = 24), 43.4 ± 8.7 years in patients admitted to our center (*n* = 11), and 23.4 ± 22.4 years in patients from the international literature (*n* = 13). Female-to-male ratio was 1.6:1. MMD predominantly affected bilateral anterior intracranial vessels. Cerebral ischemia outnumbered transient ischemic attacks (TIAs) and intracranial hemorrhage. TIAs, arterial hypertension and obesity were significantly less frequent in Southeast Asian patients compared to Caucasian Europeans. p.R4810K was absent in all examined Southeast Asians despite of typical histopathological signs of MMD in one autopsy case.

**Conclusion:**

Clinical and histopathological manifestations of MMD in Southeast Asians are similar to those in Caucasian Europeans. The genotype of MMD in Southeast Asians differs from that of most East Asian patients.

## Introduction

Moyamoya disease (MMD) is a rare, progressive, uni- or bilateral occlusion process of intracranial vessels, affecting the distal internal carotid artery (ICA) and proximal segments of the anterior (ACA) and middle cerebral artery (MCA) [1]. It is also characterized by the consecutive formation of multiple, fragile collateral networks of small caliber close to the skull base with a cloud-like angiographic appearance [2]. MMD can cause hemodynamic, embolic, and/or hemorrhagic stroke. To prevent such severe events, bypass surgery linking the superficial temporal artery to the ipsilateral MCA has been established. MMD is best known and most diagnosed in East Asia (i.e., Japan, China, and Korea). Initially, when first described by Takeuchi and Shimuzi, MMD was thought to be an exclusively Japanese disorder [3]. Incidence of MMD in East Asia ranges from 0.43 in China to 1.7–2.3 per 100,000 inhabitants per year in Korea, and prevalence is between 3.92 and 16.1 per 100,000 inhabitants [3–5]. Immigration to countries outside of East Asia does not influence the incidence of MMD although the double hit hypothesis underlines the impact of genetic and environmental factors on disease onset [6]. Epidemiologic data on patients with non-East Asian ancestry in general are rare, especially in patients of European ancestry. Furthermore, they are completely lacking in patients form Southeast Asian countries such as Cambodia, Indonesia, Laos, Malaysia, Myanmar, the Philippines, Singapore, Thailand, and Vietnam. Importantly, to our knowledge, there is no major scientific paper on the demographics and clinical presentation of MMD in Southeast Asians patients addressing the founder variant p.R4810K in the *RNF213* gene.

In East Asia, e.g., Japan and Korea, but also in China, an association of MMD with p.4810 K has been described [3]. In Japanese and Korean patients, this founder variant can be detected in 67.4–90% of typical patients [3]. In Japan, it is present in 95% of familial and 79% of sporadic cases, whereas only 1.4% of the general Japanese population carry this variant. In a meta-analysis, the founder variant p.R4810K significantly increased the risk of familial MMD in Japanese, Koreans, and Chinese [3]. In Han Chinese MMD patients, the founder variant was found in 13% [3, 7]. Although the penetrance of the variant is low and estimated to be between 1:150 and 1:300, the overall number of individuals at risk carrying the founder mutation is 38,000 for Chinese, 4500 for Korean, and 11,300 for Japanese ancestry [8–10].

Given that a post-mortem histopathological analysis of a Caucasian German patient negative for the p.R4810K variant demonstrated similar (histo-) pathological changes as in East Asian patients, we formulated the hypothesis of a common final pathway in Caucasian Europeans compared to East Asian patients, despite different genetic triggers and different clinical characteristics [11, 12].

The aim of the present study was to analyze the disease presentation, *RNF213* genetic status, and demographics of our patients with Southeast Asian origin and to compare them with Caucasian European data. Moreover, an exemplary presentation of histopathological data of one deceased member of our Southeast Asian cohort was added to support the notion that the aforementioned late-stage pathway of MMD also applies to patients of Southeast Asian origin.

## Methods

### Standard protocol approvals, registrations, and patient consents

The study was approved by the Ethics Committee of the University Hospital Düsseldorf (2019–694). All subjects were informed in detail and signed the consent for participation, in particular for the genetic testing.

### Characterization of Southeast Asian patients with MMD based in Germany

Medical records of consecutive patients of Southeast Asian ancestry in our single center database of MMD were retrospectively reviewed. All patients with MMD are followed up in the European Reference Network Center (ERN) for Moyamoya angiopathy of Alfried Krupp Hospital in Essen (Germany).

The regional classification of Asia is controversial. Mostly, East Asia includes Japan, Korea, and China. Southeast Asia geographically includes Brunei, Indonesia, Cambodia, Laos, Malaysia, Myanmar, East Timor, the Philippines, Singapore, Thailand, and Vietnam. However, in many of these Southeast Asian countries, immigrants, especially from China, and mixed ethnic ancestors are common. Patients, who themselves or their parents came from Southeast Asia, but who stated that their ancestors belonged to an East Asian minority, e.g., the Chinese minority in Thailand, Indonesia, or Singapore, were excluded from participation in the study. Patients were included in this study if all four grandparents were of Southeast Asian ethnicity and there was no evidence that their great-grandparents had an origin other than Southeast Asia. For every patient, a standardized set of demographic and clinical data were extracted by means of chart review. We referred to symptoms during the whole documented course of MMD, not only presenting symptoms.

### Review of the literature

We systematically searched articles in PubMed, using a combination of the search terms “Moyamoya” or “Moya” with one of the following search terms: “Thai”, “Thailand”, “Vietnam”, “Vietnamese”, “Indonesia”, “Indonesian”, “Cambodia”, “Cambodian”, “Brunei”, “Bruneian”, “Laos”, “Laotian”, “Malaysia”, “Malaysian”, “Myanmar”, “Burma”, “Birma”, “East Timor”, “Singapore”, “Singaporean”, “Philippines”, “Philippine”, “Southeast Asia”, and “Southeast Asian”. Patients of Chinese, other East Asian or Caucasian European descent were excluded from the analysis of Southeast Asian patients, if they were diagnosed with MMD and treated in Southeast Asia. Patients who were not diagnosed as idiopathic *Moyamoya disease* but as *Moyamoya syndrome* according to the case reports and the independent assessment of two authors (DS and MK) were excluded from the evaluation. In this context, it is important to clearly differentiate between the terms *Moyamoya angiopathy (MMA)*, *Moyamoya disease (MMD)*, and *Moyamoya syndrome (MMS)*. *Moyamoya angiopathy* is a generic term referring to a specific steno-occlusive pattern of the supraclinoid internal carotid artery and its main branches, and the development of a collateral network of fragile vessels [13–15]. The idiopathic type of MMA is called MMD. The term *Moyamoya syndrome* refers to an association of the Moyamoya phenomenon with other defined diseases, such as Down's syndrome, Neurofibromatosis type 1, alpha thalassemia, or cranial irradiation in the medical history [13]. Patients with known *Moyamoya syndrome* were excluded from participation in the study. MMD was diagnosed by means of magnetic resonance tomography angiography and digital subtraction angiography. Additionally, each patient who was diagnosed at our department underwent lumbar puncture in order to assess the likelihood of inflammatory differential diagnoses, such as varicella zoster virus vasculitis or primary vasculitis of the central nervous system. While MMA was considered as a strictly bilateral pathology in the past, since 2021, new guidelines include the unilateral phenotype as a possible manifestation of MMA [16]. Therefore, we included unilateral MMA in our study.

**Fig. 1:**
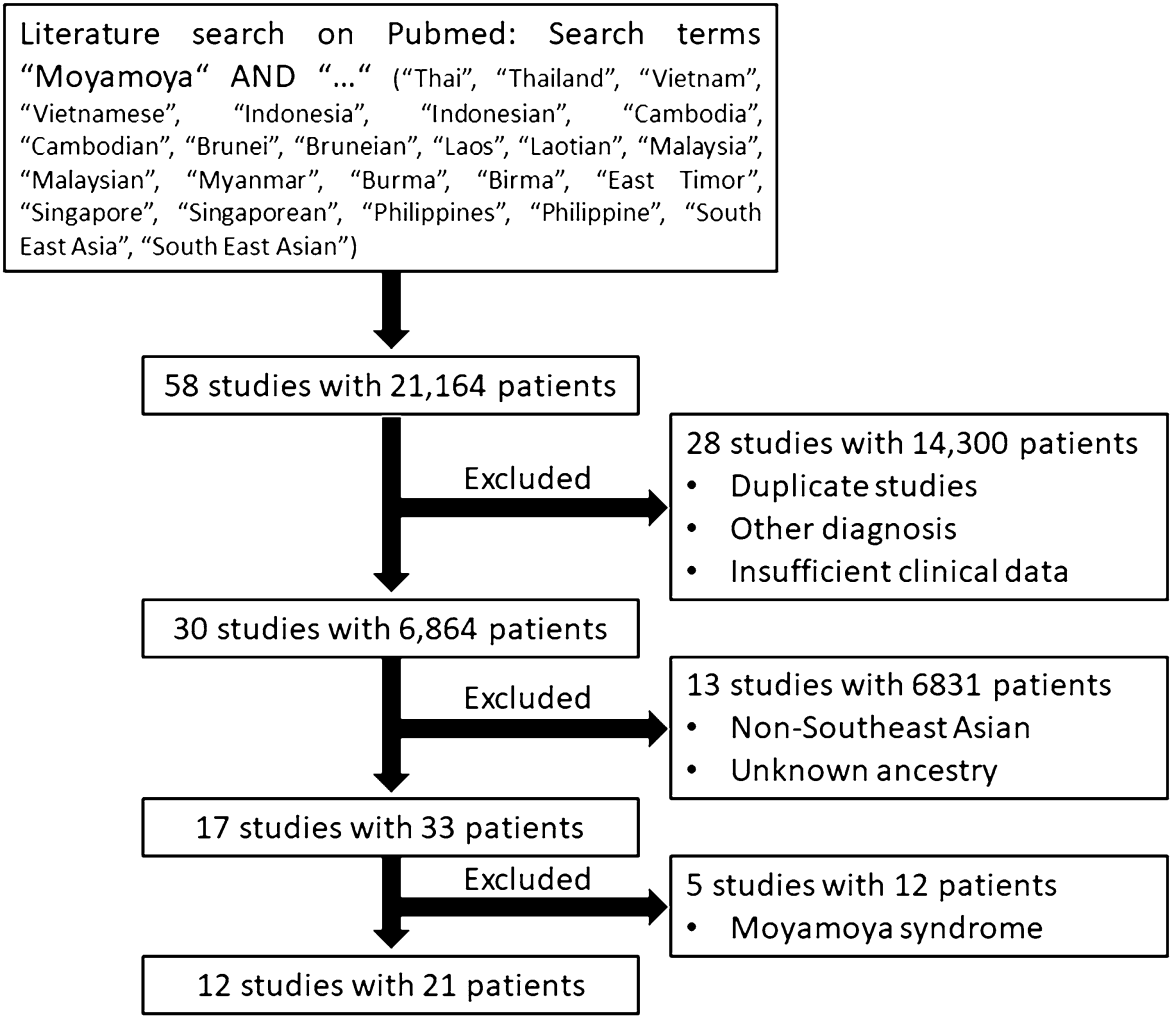
Flowchart of the literature search

A detailed description of the search strategy and study selection is depicted in Fig. 1. All identified patients were characterized using the same set of demographic, clinical, and imaging criteria as in the Southeast Asian patients based in Germany. To assess bias, we created, whenever applicable, funnel plots of the studies included in our meta-analysis, which conformed to the expected shape of the curve and demonstrated overall left–right symmetry.

### Comparison with Caucasian European data

For comparison of the Southeast Asian data, Germany-based Southeast Asian and Southeast Asian patients from the literature were pooled and compared to Caucasian Europeans published previously [12]. The statistical analysis of group differences was performed using PSPP 1.4.1. Group differences with regard to numeric variables were calculated using the Mann–Whitney U test, while dichotomous variables were analyzed by means of cross tables and Fisher's exact test. For all results, the level of significance was set to *p* < 0.05. Because this study is an exploratory analysis, we did not perform alpha adjustment.

### Genetic testing

Genetic testing was performed for *RNF213* (Ring finger protein 213) gene founder variant p.R4810K in all identified patients from our institution.

The deoxyribonucleic acid (DNA) isolation was performed from ethylenediamine tetraacetic acid (EDTA) blood using QIAsymphony DSP DNA Mini Kit (192) (Qiagen), in combination with the QIAsymphony SP instrument (Qiagen). Polymerase chain reaction (PCR) amplification was performed employing the HotStarTaq DNA Polymerase kit (Qiagen). Amplified PCR products were cleaned up using the illustra™ ExoProStar™ 1-STEP kit (VWR). The cleaned-up PCR products were cycle sequenced employing the BrilliantDye™ Terminator v1.1 Cycle Sequencing Kit (NimaGen), followed by a bead-based clean-up of the cycle PCR products and subsequent capillary electrophoresis on the 3730xl DNA Analyzer. Generated.ab1 files were analyzed with the aid of the SeqPilot software. The used oligonucleotides (primers) had the following base sequence:

131087_RNF213-E60F: ACGCTGCATCACAGGAAA

131088_NF213-E60R: GCTTAGGCTATAGAGCACCCA.

### Autopsy data and single case description

Histopathological data from post-mortem examination were collected in one deceased patient of Thai descent by one co-author (K.K.). The medical history of this patient is described exemplarily. Histological sections were stained with hematoxylin & eosin, Berlin blue, Elastica van Gieson stain, and immunohistochemically with an antibody against smooth muscle actin.

## Results

### Characteristics of 11 Southeast Asian patients based in Germany

Eleven Southeast Asian patients with MMD were admitted to the European Reference Network Center (ERN) for Moyamoya angiopathy of the Alfried Krupp Hospital in Essen (Germany); eight (72.7%) of whom were female (see Table 1). Mean age was 43.4 ± 9.2 years at onset (range 31–61 years) and 45.7 ± 10.1 at diagnosis (range 31.2–61.3 years). The patients under investigation were of Vietnamese (*n* = 5), Thai (*n* = 4), and Indonesian (*n* = 2) descent, and all had adult-onset disease. MMD was almost exclusively bilateral (*n* = 10) and confined to the anterior intracranial vasculature (see Table 1). Cerebral ischemia (63.6%) and TIA (27.3%) were more frequent than cerebral bleedings (18.2%). One patient (9.8%) experienced seizures. 45.5% were affected by cognitive/psychiatric deficits, while three individuals had suffered episodes of syncope. Most patients were exposed to at least one cardiovascular risk factor (see Table 1). None of the patients had a positive genetic screening for the East Asian MMD founder variant (p.R4810K) of the *RNF213* gene (see Table 1). Patient 3 deceased and her autopsy data are described later.

**Table 1:** Characterization of Southeast Asian patients based in Germany

Demographics	Patient # 1–2	Patient # 3–6	Patient # 7–11	Overall *N* (%)
Origin	Indonesia	Thailand	Vietnam	
Male	0	1	2	3 (27.3)
Female	2	3	3	8 (72.7)
Age of onset, mean (± SD)	53.4	41.6	40.8	43.4 (9.2)
Age of onset, median (range)	53.4	41.4	37.1	43.2 (31–61)
Age of diagnosis, mean (± SD)	61.3	42.3	42.3	45.7 (10.1)
Age of diagnosis, median (range)	61.3	42.0	43.6	44.1 (31.2–61.3)
Children at onset	0	0	0	0 (0.0)
Adults at onset	2	4	5	11 (100.0)
Family history	0	0	1	1 (9.1)
Vascular features				
Unilateral	0	0	1	1 (9.1)
Bilateral	2	4	4	10 (91.0)
P1 stenosis	1	2	0	3 (27.3)
Vertebrobasilar impairment	1	0	0	1 (9.1)
Extracranial impairment	1	1	0	2 (18.2)
Intracranial aneurysm	0	0	0	0 (0.0)
Symptoms				
TIA/limb shaking TIA	1	0	2	3 (27.3)
Cerebral ischemia	2	3	2	7 (63.6)
Cerebral hemorrhage	0	0	2	2 (18.2)
Epilepsy	0	1	0	1 (9.1)
Syncope	0	2	1	3 (27.3)
Cognitive/psychiatric	0	2	3	5 (45.5)
Retinal stroke	0	0	1	1 (9.1)
Associated features				
Cardiac illness	0	0	1	1 (9.1)
Arterial hypertension	2	1	3	6 (54.5)
Adiposity	1	0	0	1 (9.1)
Diabetes mellitus	1	3	1	5 (45.5)
Thyroid abnormalities	1	2	2	5 (45.5)
Genetic abnormalities				
Negative for *RNF213*p.R4810K	2	4	5	11 (100.0)

### Characteristics of reported Southeast Asian patients with MMD from the international literature

Twenty-one cases of MMD in Southeast Asian patients were extracted from the already existing body of international literature [17–28]. Twelve patients (57.1%) were female (see Table 2). Mean age was 23.4 ± 22.5 years at onset (range 2–68 years) and 20.2 ± 20.3 at diagnosis (range 3–81 years). The inversion of the age of onset and diagnosis is due to missing values. The patients were of Thai (*n* = 15), Malaysian (*n* = 2), Vietnamese (*n* = 1), Indonesian (*n* = 1), and Singaporean (*n* = 1) descent. One Southeast Asian patient’s origin was not further specified. In 14 (66.7%) cases, MMD began in childhood. There were no records of a positive family history of MMD (see Table 2). Again, almost all patients were affected by bilateral MMD without signs of involvement of the posterior or extracranial circulation. Cases of cerebral ischemia (76.9%) outnumbered TIAs (15.4%) and intracranial hemorrhage (23.1%). Out of 19 patients with detailed descriptions of the symptoms of MMD, 11 (57.9%) suffered from seizures as the most frequent clinical concomitant of the disease. In 15 patients, no cardiovascular risk factors were reported (see Table 2).

**Table 2:** Characterization of Southeast Asian patients from the international literature

Demographics	Gunawan et al. 2017	Ng et al. 1995	Bolem et al. 2020	Tan et al. 2003	Sharma et al. 2014	Khanjanasthiti et al. 1979	Dechakaisaya et al. 1979	Visudhiphan et al. 1989
Origin	Indonesia	Malaysia	Malaysia	Singapore	Southeast Asia	Thailand	Thailand	Thailand
Male	1	1	0	0	0	0	1	3
Female	0	0	1	1	1	1	0	1
Age of onset, mean (± SD)	3	14	21	57	39	43	41	3.8
Age of onset, median (range)	3	14	21	57	39	43	41	2.5
Age of diagnosis, mean (± SD)	8	14	21	58	40	43	42	6
Age of diagnosis, median (range)	8	14	21	58	40	43	42	4.5
Children at onset	1	1	0	0	0	0	0	4
Adults at onset	0	0	1	1	1	1	1	0
Family history	n/a	0	n/a	n/a	n/a	n/a	0	0
Vascular features								
Unilateral	0	0	0	0	0	1	0	0
Bilateral	1	1	1	1	1	0	1	4
P1 stenosis	1	0	0	0	0	0	0	0
Vertebrobasilar impairment	0	0	0	0	0	0	0	0
Extracranial impairment	0	0	0	0	0	0	0	0
Intracranial aneurysm	0	0	0	0	0	0	0	0
Symptoms								
TIA	1	1	0	0	0	0	0	0
Cerebral ischemia	1	1	1	1	1	0	0	4
Cerebral hemorrhage	0	1	0	0	0	1	1	0
Epilepsy	n/a	0	0	0	0	1	1	4
Syncope	n/a	0	0	0	0	0	0	0
Cognitive/psychiatric	n/a	0	0	0	0	0	0	0
Retinal stroke	n/a	0	0	0	0	0	0	0
Associated features								
Cardiac illness	n/a	0	0	0	0	0	1	0
Arterial hypertension	n/a	0	0	0	0	0	1	0
Adiposity	n/a	0	0	0	0	0	0	0
Diabetes mellitus	n/a	0	1	1	0	0	0	0
Thyroid abnormalities	n/a	0	0	0	0	0	0	0

Demographics	Visrutaratna et al. 2009	Phattranonuthai et al. 2020	Thampratanku et al. 2022	Lubman et al. 2003	Available data n	Overall *n* (%)
Origin	Thailand	Thailand	Thailand	Vietnam		
Male	1	1	0	1	21	9 (42.9)
Female	0	0	7	0	21	12 (57.1)
Age of onset, mean (± SD)	3	68	n/a	n/a	13	23.4 (22.4)
Age of onset, median (range)	3	68	n/a	n/a	13	14 (2–68)
Age of diagnosis, mean (± SD)	5	81	9.3	23	21	20.2 (20.3)
Age of diagnosis, median (range)	5	81	9.6	23	21	11.9 (3–81)
Children at onset	1	0	7	0	21	14 (66.7)
Adult at onset	0	1	0	1	21	7 (33.3)
Family history	n/a	n/a	0	n/a	13	0 (0.0)
Vascular features						
Unilateral	n/a	0	n/a	0	14	1 (7.1)
Bilateral	n/a	1	1	1	14	13 (92.9)
P1 stenosis	n/a	0	n/a	0	13	1 (7.7)
Vertebrobasilar impairment	n/a	0	n/a	0	13	0 (0.0)
Extracranial impairment	n/a	0	n/a	0	13	0 (0.0)
Intracranial aneurysm	n/a	0	n/a	0	13	0 (0.0)
Symptoms						
TIA	n/a	0	n/a	0	13	2 (15.4)
Cerebral ischemia	n/a	1	n/a	0	13	10 (76.9)
Cerebral hemorrhage	n/a	0	n/a	0	13	3 (23.1)
Epilepsy	n/a	0	5	0	19	11 (57.9)
Syncope	n/a	0	0	0	19	0 (0.0)
Cognitive/psychiatric	n/a	0	1	1	19	2 (10.5)
Retinal stroke	n/a	0	0	0	19	0 (0.0)
Associated features						
Cardiac illness	0	0	0	0	20	1 (5)
Arterial hypertension	1	1	0	0	20	3 (15)
Adiposity	0	0	0	0	20	0 (0.0)
Diabetes mellitus	0	1	0	0	20	3 (15)
Thyroid abnormalities	0	0	0	0	20	0 (0.0)

### Comparative analysis between Southeast Asian and Caucasian Europeans with MMD

The age of diagnosis in the entire group of Southeast Asian patients (*n* = 32) was significantly lower than in Caucasian Europeans (*p* = 0.045), which is closely linked to the higher number of minors in the Southeast Asian cohort at the time of diagnosis (*p* < 0.001). Other demographic parameters did not show any marked differences (see Table 3). TIAs were much more frequent in Caucasian Europeans than in Southeast Asians (*p* < 0.001). Arterial hypertension (*p* = 0.033) and adiposity (*p* = 0.002) were detected significantly more often in Caucasian Europeans than in Southeast Asians (see Table 3). The opposite was true for diabetes mellitus (*p* = 0.036), which was type 2 in all but one.

**Table 3:** Comparison of demographic, neuroradiological, and clinical features

Demographics n (%)	Southeast Asians*	Caucasian Europeans**	*P* value
All patients	32 (100.0)	200 (100)	
Male	12 (37.5)	48 (24)	0.128
Female	20 (62.5)	152 (76)	
Age of onset, mean (± SD)	32.5^a^ (± 20.3)	32.9^e^ (14)	0.769
Age of onset, median (range)	38.1 (2–68)	32^e^ (1–71)	
Age of diagnosis, mean (± SD)	29.0 (± 21.3)	36^e^ (14.1)	0.045
Age of diagnosis, median (range)	27.1 (3–81.0)	35^e^ (3–71)	
Children at onset	14 (43.8)	25^e^ (12.7)	< 0.001
Adults at onset	18 (38.1)	172^e^ (87.3)	
Family history	1^a^ (6.5)	11^f^ (5.7)	1.000
Vascular features			
Unilateral	2^b^ (8)	51 (25.5)	0.077
Bilateral	23^b^ (92)	149 (74.5)	
P1 stenosis	4^a^ (16.7)	23^ g^ (11.6)	0.505
Vertebrobasilar impairment	1^a^ (4.2)	14^ g^ (7.0)	1.000
Extracranial impairment	2^a^ (8.3)	35^ g^ (17.6)	0.384
Intracranial aneurysm	0^a^	8 (4)	1.000
Symptoms			
TIA/Limb shaking TIA	5^a^ (20.8)	143 (71.5)	< 0.001
Cerebral ischemia	17^a^ (70.8)	164 (82)	0.269
Cerebral hemorrhage	5^a^ (20.8)	19 (9.5)	0.151
Epilepsy	12^c^ (40)	61 (30.5)	0.300
Syncope	3^c^ (10)	13 (6.5)	0.446
Cognitive/psychiatric	7^c^ (23.3)	41 (20.5)	0.810
Retinal stroke	1^c^ (3.3)	3 (1.5)	0.431
Associated features			
Cardiac illness	2^d^ (6.5)	12 (6)	1.000
Arterial hypertension	9^d^ (29)	100^ g^ (50.2)	0.033
Adiposity	1^d^ (3.2)	54^e^ (27.4)	0.002
Diabetes mellitus	8^d^ (25.8)	21^ h^ (10.6)	0.036
Thyroid abnormalities	5^d^ (16.1)	35^i^ (23.8)	0.479

Due to missing data, the cohort was smaller than indicated in line one:

^a^n = 24, ^b^n = 25, ^c^n = 30, ^d^n = 31, ^e^n = 197, ^f^n = 194, ^g^n = 199, ^h^n = 198, ^i^n = 147

^*^Data of 11 German based and 21 Southeast Asian patients from literature,

^**^ Data were adapted from Kraemer et al. 2019

### Autopsy finding and single case description

In April 2021, the initial diagnosis of a bilateral MMD was made in an adult patient, who originally immigrated to Germany from Thailand. The patient had previously suffered bilateral MCA infarctions and right-sided ACA stroke. Recent cerebral imaging showed advanced bilateral MMD with bilateral occlusion of the proximal MCA and collateral formations characteristic of MMD. Due to recurrent strokes, the patient was treated with aspirin 100 mg and clopidogrel 75 mg daily. The medical history was positive for diabetes mellitus, arterial hypertension, and hepatopathy triggered by hepatitis E infection and exacerbated by medication with levetiracetam and statins. Clinically, the patient suffered from global aphasia, dysphagia and encephalopathic signs. Eight days before the first bypass surgery, clopidogrel was discontinued, while aspirin was continued. The patient underwent bypass surgery for the first time on the left in July 2021 in Alfried Krupp hospital, Germany, without any complications. In February 2022, cerebral bypass surgery on the right took place. Immediate postoperative course was normal. Again, discontinuation of clopidogrel was requested 8 days before surgery and continuation of aspirin was agreed upon. Cerebral computed tomography on the first postoperative day did not show any intracranial bleeding or new ischemia. Due to the stable clinical and imaging state, the patient was treated at the normal ward. On the third postoperative day, the patient was pale and lifeless reviving with immediate resuscitation. Cerebral computed tomography depicted a pronounced subdural hemorrhage on the right with a clear midline shift to the left. Given the unfavorable prognosis based on wide, fixed, and unrounded pupils, palliative care was started. The patient developed a septic shock and died within the next 24 h. On the day of death, it was detected by laboratory chemistry that the patient still had a slight effect of clopidogrel in the blood in addition to the effect of aspirin. A connection with the previously detected but normalized hepatopathy can be hypothesized.

**Fig. 2:**
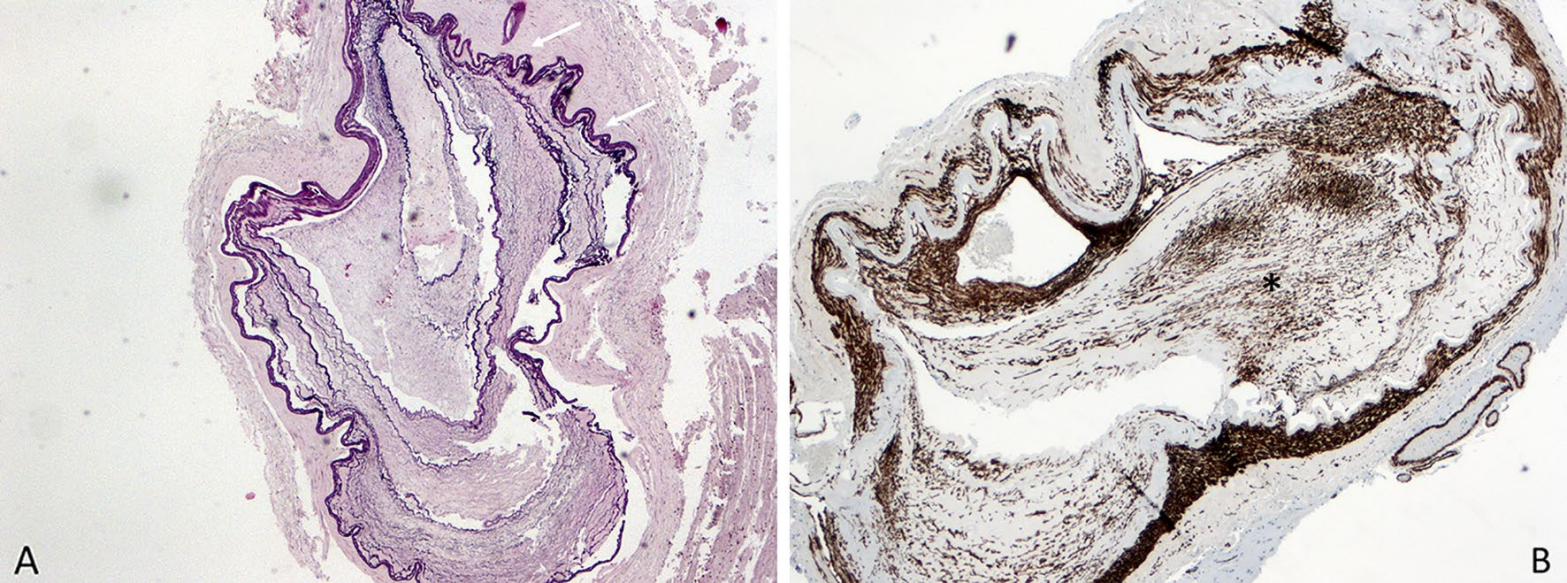
Histopathological staining of the internal carotid artery of a deceased female Thai Moyamoya patient: **A** high-grade Moyamoya pathology in the form of thickening and undulation and partly Duplication or triplication of the internal elastic membrane (white arrow) (Elastica van Gieson staining); **B** fibrocellular thickening and smooth muscle cell proliferation of the tunica intima (actin staining) (black star)

Histopathologic post-mortem examination of the cerebral and brain-supplying vasculature revealed atherosclerotic changes of the vertebral artery on both sides, basilar artery, left posterior cerebral artery, posterior communicating artery on the right and ICA (segments C1–C5) on both sides in the form of widening and focal disruption of the internal elastic membrane and fibroelastosis of the tunica media and partial calcifications. In the distal segment of the ICA (segments C6 and C7), bilateral mild, in the proximal and middle segments of the anterior (A1-2) and middle (M1-2) cerebral arteries on both sides, high-grade Moyamoya pathology in the form of an undulation and partly duplication or triplication of the internal elastic membrane (Elastica van Gieson staining, see Fig. 2a) as well as fibrocellular thickening and proliferation of smooth muscle cells of the tunica intima (actin staining, see Fig. 2b) were detected. Further typical histopathological features of MMD, such as a fragmented elastic lamina, fibrin deposits in the vessel wall, and an attenuated tunica media were detected as well, while no microaneurysms could be found. Moreover, multiple stage III infarctions, pronounced acute subdural hematoma, high-grade hypoxic–ischemic encephalopathy, and increased intracranial pressure with congestive bleeding in the brainstem were present and closely related to the patient’s sudden death.

## Discussion

In recent years, the disease presentation in Moyamoya patients of ethnic origin outside East Asia has been increasingly studied, especially in Caucasian Europeans and Americans [12, 29, 30]. It still remains a matter of debate, whether MMD has different pathophysiological, clinical, and diagnostical features in other ethnicities than in East Asians [31, 32]. In particular, the susceptibility variant *RNF213* p.R4810K is considered relevant in East Asians in contrast to Caucasian Europeans [10, 33, 34]. Accordingly, the question arises whether the disease should be treated in other ethnicities according to the same principles as in East Asians. For example, data from the Japanese AMORE study on stroke incidence in asymptomatic patients with MMD showed a stroke rate of 1.4% per person in the first five years [35]. The issue at hand is whether these findings, consisting of six hemorrhagic and one ischemic stroke in 103 Japanese patients, are applicable to populations of different ethnicities, who are believed to have lower risk of bleeding [31]. The first European guidelines on the disease were based mainly on East Asian data, given the scarcity of data in Caucasian European patients [13]. Despite differences in the genetic predisposition and some differences in clinical presentation, histopathology, and most clinical symptoms in Caucasian Europeans resemble those in East Asians [11, 13].

In our study, we felt that it was crucial to solely enroll patients who self-identified and traced their heritage to Southeast Asia, despite acknowledging the contentious and intricate nature of ethnicity in epidemiological research [36].

In 1992, Goto and colleagues described the worldwide spread of MMD for the first time. However, four cases from Thailand and two cases from Malaysia were included without further details [37].

As shown in our present analysis, the demographic and clinical presentation of MMD in Southeast Asians appears similar to the disease presentation in Caucasian Europeans. Comparison of most parameters between the 32 Southeast Asian patients analyzed here with a cohort from the literature of 200 Caucasian Europeans from the literature showed no differences [12, 38]. The significant statistical difference in the number of children at the onset of the disease may be biased by the fact that only adults were treated at our German center and that many cases of children were reported in the international literature on Southeast Asians.

Methodologically, it was not possible to compare the data presented here from Southeast Asians with East Asians from Japan and Korea. The National Registry in Japan only registers the presenting symptom and not the frequency of symptoms of affected individuals in general. In this respect, the comparison to Caucasian Europeans was made, because the study design was comparable. Nevertheless, if we look at the data of East Asians with the methodological limitations, the following is striking:

In the national registry of Japanese patients with MMA, ischemic stroke occurred as presenting symptom in 29.4% of all patients, followed by TIAs in 29.1% and a traditionally relatively high proportion of intracranial hemorrhage of 25.3% [38]. In another Japanese cohort, a similar proportion of hemorrhages of 20% has been found, occurring most frequently within the first 30–39 years of the patients’ life spans [3, 4]. In the latter study, the percentage of cerebral ischemia was 57.4%, with two age peaks around the 5th and 9th year of age and between the 45th and 49th year of age [3, 4]. Members of our cohorts, especially Southeast Asian patients, exhibited much more ischemic strokes (70.8% in Southeast Asian and 82% in Caucasian European patients). This underlines the hypothesis that East Asian patients with MMD are more likely to suffer an intracerebral hemorrhage than European Caucasians [12, 39]. Of note, data are inconsistent with cerebral hemorrhage occurring in 0% of pediatric patients with MMA in Italy, 8.5 to 9.5% in Germany, 15.1% in Italian adults, and 23% in the (mainly) Danish cohort [12, 40–43].

Given the demographic discrepancies between Southeast Asians and Caucasian Europeans in our study, the significant differences in the proportion of arterial hypertension and obesity may result from the differences in the patients’ age. It is more likely to accumulate more cardiovascular risk factors with increasing age. Additionally, the quality and accessibility of medical care in the respective countries exercise a strong influence on the detection of such risk factors.

It is probable that certain aspects and incidents, such as transient ischemic attacks (TIAs), are not accurately documented in nations with predominantly general practitioner-led and rudimentary healthcare systems. It is also likely that in countries like Germany, a lesser known disease such as MMD is often overlooked, misdiagnosed, or underdiagnosed [44]. Moreover, the healthcare and social status of individuals with a history of migration, such as those from Southeast Asia residing in European countries, can affect the precision of diagnoses and accessibility of healthcare resources. This might also bias our data.

Therefore, certain morbidities might be underrecognized in less-developed areas of Southeast Asia. Of note, the higher proportion of diabetes mellitus in Southeast Asians does not seem to fit into this causal chain. Given that five out of eight Southeast Asians with diabetes mellitus (62.5%) were diagnosed and treated in Germany, a certain convergence of (acquired) risk profiles might have occurred. Due to the total number of 32 included Southeast Asians, even slight differences in frequency of certain attributes can massively influence proportions.

With 11 cases analyzed, the study presented here is the largest to date to systematically analyze the founder variant *RNF213* p.R4810K in Southeast Asian patients with MMA. In a population-based study, Liu and colleagues found the *RNF213* p.R4810K variant in germinal cases in 103 Vietnamese healthy volunteers and 50 Filipino healthy volunteers [8]. However, it remained unclear whether this variant also plays a role in Southeast Asia due to the rarity of the disease and the fact that Liu and colleagues analyzed healthy volunteers only.

Absence of the *RNF213* p.R4810K variant in MMD was reported by Shoemaker in two Vietnamese patients, one patient from Cambodia, one from Laos, and one from Malaysia, which is consistent with our data. This supports the assumption that the variant *RNF213* p.R4810K is not present in Southeast Asians with MMD. In this respect, we assume a common pathophysiological final pathway, although we could show here for the first time that the variant *RNF213* p.R4810K is not associated with MMD in Southeast Asians.

Our study is the first to describe histopathological findings of MMD in a Southeast Asian patient. The main pathological hallmarks of MMD, including thickening and undulation of the internal elastic lamina, fibrocellular thickening, and proliferation of smooth muscle cells of the intima of the affected vascular segments, were in accordance with findings in East Asian patients and a German patient with MMD, thereby supporting the hypothesis of a common final cerebrovascular manifestation despite different genetic predisposition and possibly different disease onset [17, 45, 46].

The data suggest that MMD in Southeast Asians is clinically similar and can be treated in a similar way as in Caucasian Europeans or Americans. However, larger studies are needed, especially with comparable strategies of data collection and comparison to Japanese and Koreans. As long as the National Moyamoya Registry in Japan only collects presenting symptoms, no comparative statement can be made between Japanese, Caucasian Europeans or Americans, and Southeast Asians on the general symptoms in the course of the disease [38].

Overall, it is important to think of MMD in the case of unexplained strokes at young age in patients with Southeast Asian ancestry. More research should be done to study the disease in more detail in patients of this origin.

## Data access

MK takes full responsibility for the data, the analyses and interpretation, and the conduct of the research. The authors have full access to all of the data and have the right to publish any and all data, separate and apart from the guidance of any sponsor.

## Data Availability

Anonymized data not published within this article will be made available by request from any qualified investigator.
